# STANDARDIZED CLINICAL PATHWAYS FOR ESOPHAGECTOMY ARE NOT A REALITY IN BRAZIL, EVEN WITH A HIGH PREVALENCE OF ESOPHAGEAL CANCER AND ACHALASIA

**DOI:** 10.1590/S0102-67202015000300011

**Published:** 2015

**Authors:** Marina ZAMUNER, Fernando A. M. HERBELLA, José L. B. AQUINO

**Affiliations:** 1 Department of Surgery, University of Campinas, Campinas, SP; 2 Department of Surgery, School of Medicine, Federal University of São Paulo, São Paulo,SP, Brasil

**Keywords:** Esophagectomy, Perioperative care, Clinical pathways, Outcomes, Multidisciplinary team

## Abstract

**Background:**

The adoption of standardized protocols and specialized multidisciplinary teams for esophagectomy involve changes in routines with the implantation of expensive clinical practices and deviations from ingrained treatment philosophies.

**Aim:**

To evaluate the prevalence of standardized protocols and specialized multidisciplinary teams in São Paulo state, Brazil.

**Methods:**

Institutions that routinely perform esophagectomies in São Paulo were contacted and questioned about the work team involved in the procedure and the presence of standardized routines in the preoperatory care.

**Results:**

Fifteen centers answered the questionnaire: 10 (67%) public institutions and five (33%) private. There were seven (47%) medical schools, six (40%) with a residency program and two (13%) nonacademic institutions. The mean number of esophagectomies per year was 23. There was a multidisciplinary pre-operative team in nine (60%). There was a multidisciplinary postoperative team in 11 (73%). Early mobilization protocol was adopted in 12 (80%) institutions, early feeding in 13 (87%), routinely epidural in seven (47%), analgesia protocol in seven (47%), hydric restriction in six (40%), early extubation in six (40%), standardized hospitalization time in four (27%) and standardized intensive care time in two (13%).

**Conclusion:**

The prevalence of standardized protocols and specialized teams is very low in Sao Paulo state, Brazil. The presence of specialized surgeons is a reality and standardized protocols related directly to surgeons have higher frequency than those related to other professionals in the multidisciplinary team.

## Abstract (pt)

**Racional:**

A adoção de protocolos padronizados por equipe multidisciplinar especializada no perioperatório de esofagectomia melhora a morbimortalidade da operação, porém envolve implantação de práticas por vezes custosas e mudanças de rotinas e filosofias arraigadas.

**Objetivo:**

Avaliar a ocorrência de protocolos padronizados e equipe multidisciplinar para esofagectomia no estado de São Paulo.

**Métodos:**

Foram contactadas instituições que realizam esofagectomias rotineiramente e questionadas a respeito da equipe envolvida no procedimento e a ocorrência de rotinas clínicas padronizadas no perioperatório dos pacientes.

**Resultados:**

Das 15 instituições respondedoras eram 10 (67%) públicas e cinco (33%) privadas; sete (47%) escolas médicas, seis (40%) com programa de residência e duas (13%) não acadêmicas. Estas realizavam em média 23 esofagectomias por ano. Nove (60%) instituiçoes possuíam equipe multidisciplinar especializada no pré-operatório e 11 (73%) no pós-operatório. Devido a existência de protocolos, foram adotados: mobilização precoce em 12 instituições (80%); alimentação precoce em 13 (87%); epidural rotineira em sete (47%), protocolo de analgesia em sete (47%), restrição hídrica em seis (40%), extubação precoce em seis (40%), tempo de hospitalização padrão em quatro (%) e tempo de UTI padrão em duas (13%) instituições.

**Conclusão:**

É baixa a ocorrência de protocolos padronizados e equipes multidisciplinares especializadas para esofagectomia no estado de São Paulo. Observa-se elevada prevalência de cirurgiões especializados e maior frequência de protocolos relacionados diretamente aos cirurgiões, em detrimento aos outros profissionais da equipe multidisciplinar.

## INTRODUCTION

Esophageal cancer is a devastating disease. Survival is dismal and inferior to other tumors. Earlam and Cunha-Melo 3 reviewed literature earlier to 1980 to show a 10% 5-year survival among patients submitted to esophagectomy. Currently, results as good as 64% - but still suboptimal - may be obtained with extensive radical operations 19 ; however, these outcomes have not been significantly improved in the last years. Probably current available therapy reached its maximum and new forms of treatment are expected. 

Surgery has been considered an essential part of the treatment of patients with esophageal carcinoma; however, better survival achieved with surgical therapy has paid a high price. Esophagectomy is a technically demanding and complex operation with high rates of morbidity and mortality. In 1980, Earlam and Cunha-Melo 1 again reviewed the literature and reported 29% mortality rate for esophagectomy. Modernly, 22% mortality rate is still reported 2 . This data brings the question if survival for esophageal cancer is it a matter of dying by the cancer or dying by the knife.

The outcomes for esophageal resection seem to be influenced by the adoption of standardized protocols 4 and specialized multidisciplinary teams 17 .

Esophageal cancer in the state of São Paulo, Brazil is the 6^th^ neoplasia in men, corresponding to 2.7% of all malignancies in the state 10 .

Achalasia secondary to Chagas disease is also a health problem. Although the number of autochthonous cases from São Paulo is small, migration from other areas of the country for treatment is very common. Esophagectomy is one of the therapies proposed for dilated megaesophagus which represents a significant number of the cases 9 .

This study aims to evaluate the prevalence of the implementation of standardized perioperative routines for esophagectomy in the state of São Paulo, Brazil.

## METHODS

The study was approved by the Institutional Review Board under number 288.432/2013. 

Institutions in the State of São Paulo that routinely perform esophagectomy, for benign or malign disease, were contacted and questioned about the team involved in the process and the implementation of standardized perioperative routines.

The selection of the contributors was made considering recent publications in the field, participation in meetings, networks and indication of participants. There is no official registration of esophagectomies in Brazil. 

### Questionnaire

A senior team member were contacted by e-mail or phone, and questioned about: 1) the annual number of esophagectomies performed in the institution; 2) the existence of a specialized surgical team; 3) the presence of a specialized anesthesiologist; 4) the presence of a multidisciplinary pre and postoperative team and its members; 5) the existence of standardized protocols, such as hydric restriction, early extubation, analgesia, routinely epidura, early deambulation, feeding, intensive care time and hospitalization time.

Fisher or Mann-Whitney tests were used when appropriate for statistical analysis and p<0.050 was considered signiﬁcant. 

## RESULTS

Seventeen institutions were contacted, 15 (88%) answered the questionnaire. Among those that answered, 10 (67%) were public institutions and 5 (33%) private. There were 7 (47%) medical schools, 6 (40%) institutions with a residency program and 2 (13%) nonacademic institutions. 

**FIGURE 1:**
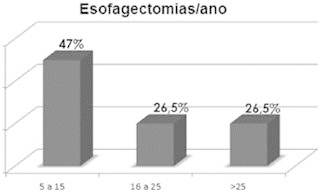
Annual rate of esophagectomy per year for the participant institutions

The mean number of esophagectomies per year was 23±18 (range 5-60) ( Figure 1 ).

Thirteen (87%) institutions had specialized surgical team and four (27%) specialized anesthesiologist. 

There was a multidisciplinary pre-operative team in nine (60%) institutions; counting with surgeon in nine (100% of those with a multidisciplinary team); oncologist in seven (78%), nutritionist in six (67%), physiotherapist in five (56%) anesthesiologist in two (23%), nurse in three (33%), psychologist in two (33%), endoscopist in two (23%), pulmonologist in one (11%), cardiologist in one (11%) and pathologist in one (11%). 

There was a multidisciplinary postoperative team in 11 (73%) institutions; counting with surgeon in 11 (100% of those with a multidisciplinary team), oncologist in nine (82%), physiotherapist in eight (73%), nutritionist in seven (64%), radiotherapist in four (36%), nurse in three (30%), psychologist in three (18%), pathologist in two (18%), anesthesiologist in two (18%), endoscopist in one (9%) and audiologist in ome (9%).

Early mobilization protocol was adopted in 12 (80%) institutions; early feeding in 13 (87%); routinely epiduralin seven (47%); analgesia protocol in seven (47%); hydric restriction in six (40%); early extubation in six (40%); standardized hospitalization time in four (27%) and standardized intensive care time in two (13%).

Table 1 shows the correlation between the number of esophagectomies per year and others variables, and Table 2 the correlation between public and private institutions and other variables. There were no differences between the groups. 

**TABLE 1:** Correlation between the number of esophagectomies per year and others variables

Esophagectomy/year	5 to 15 (n=7)	15 to 25 (n=4)	More than 25 (n=4)	p value
Public	57%	50%	50%	1
Hydric restriction	0%	25%	100%	1
Early extubation	28%	50%	50%	1
Analgesia	14%	75%	75%	1
Epidural	43%	50%	50%	1
Early mobilization	71%	75%	100%	1
Early feeding	71%	100%	100%	1
Surgical team	71%	100%	100%	1
Anesthesiologist	14%	0%	75%	1
Pre-operatory team	43%	75%	75%	1
Postoperative team	57%	75%	100%	1

**TABLE 2:** Correlation between public and private institutions and other variables

Public institutions (n=10)	Private Institutions (N=5)	p value
Surgical team	80%	100%	1
Specialized anesthesiologist	20%	40%	0.6027
Pre-operatory team	50%	80%	0.6785
Postoperative team	70%	80%	1
Hydric restriction protocol	30%	60%	0.6311
Early extubation	40%	40%	1
Analgesia	50%	40%	1
Epidural	50%	40%	1
Early mobilization	80%	80%	1
Early feeding	80%	100%	1
Intensive care time	10%	20%	1
Hospitalization time	20%	40%	0.6027

## DISCUSSION

The outcomes for esophagectomy must not be only measured by mortality and survival 13 . The procedure is also linked to a high rate of morbidity, prolonged ICU and in-hospital time. As mentioned before, the outcomes seem to be influenced by the adoption of multidisciplinary care pathways. However, these results show a low prevalence of implementation of standardized protocols for esophagectomy in the state of São Paulo.

It seems to have a clear direct relation between the volume of esophagectomies and outcomes 18 . The annual rate of procedures probably influences not only surgeon's expertise but also the multidisciplinary team experience. In our results, even though standardized protocols and specialized teams were more prevalent in high volume centers, statistical significance was not reached. This fact may reflect the small number of included institutions. It seems intuitive that the adoption of standardized protocols may be more difficult in low volume centers; however, most available series come from centers reporting results from less than seven esophagectomies/year 1 4 11 12 15 .

Even though our report does not evaluate outcomes, the adoption of standardized protocols and multidisciplinary care seems to improve outcomes and thus may be considered an improvement in care, especially in countries with a high prevalence of esophageal cancer and achalasia. Findlay et al. 4 recently reviewed the topic and found that five series reported reductions in length of stay; one reported reductions in pulmonary complications, mortality, and length of stay; and two reported reduction in complications overall. The benefits of standardized clinical pathways was confirmed by two metanalysis 5 14 and a prospective study 6 . 

There are major difficulties in the introduction of new clinical evidence-based guidelines into clinical practice 7 . Most of the esophagectomy protocols and the creation of tumor boards involve changes in routines with the implantation of expensive clinical practices and deviations from ingrained treatment philosophies, although the decrease of complications and length of stay may decrease costs 11 . Thus, the implementation of standardized protocols for esophagectomy can be challenging, especially in underdeveloped countries. In fact, Findlay et al. 4 reported that less than half of the patients completed the proposed pathway mostly due to the occurrence of complications. The small number of published series also attests the low prevalence of adoption of these protocols. 

Our results show that surgeons are the most specialized staff member and still the leader of the multidisciplinary team. Less than 30% had a specialized anesthesiologist, even with a well-established relationship between intraoperative anesthetic management and postoperative results 9 . Other specialties make part of the team sporadically. It has been shown that an esophagectomy-specific multidisciplinary care may lower operative mortality (5.7% vs. 26%) and increase five years survival 16 . Excluding early mobilization and feeding, standardized protocols were infrequently found in the queried institutions. 

There are limitations in this paper. This report studied a small number of institutions. It did not contemplate the entire country due to its heterogeneity. Since the state of São Paulo has the larger number of esophagectomies per year, it was presumable that it would have the best results in perioperative care matter. Also, the study did not evaluate outcomes. 

## CONCLUSIONS

The prevalence of standardized protocols and specialized teams is very low in São Paulo. The presence of specialized surgeons is a reality and standardized protocols related directly to surgeons have higher frequency than those related to other professionals in the multidisciplinary team.
